# Response to ‘promising advances and remaining challenges in AI-driven QTc estimation’ by Zekai Yu

**DOI:** 10.1093/europace/euag043

**Published:** 2026-03-24

**Authors:** Lucas Plagwitz, Florian Doldi, Jannes Magerfleisch, Maxim Zotov, Lucas Bickmann, Dominik Heider, Julian Varghese, Lars Eckardt, Antonius Büscher

**Affiliations:** Institute of Medical Informatics, University of Münster, Albert-Schweitzer-Campus 1/Building A11, Münster 48149, Germany; Clinic for Cardiology II: Electrophysiology, University Hospital Münster, Albert-Schweitzer-Campus 1/Building A1, Münster 48149, Germany; Clinic for Cardiology II: Electrophysiology, University Hospital Münster, Albert-Schweitzer-Campus 1/Building A1, Münster 48149, Germany; Institute of Medical Informatics, University of Münster, Albert-Schweitzer-Campus 1/Building A11, Münster 48149, Germany; Institute of Medical Informatics, University of Münster, Albert-Schweitzer-Campus 1/Building A11, Münster 48149, Germany; Institute of Medical Informatics, University of Münster, Albert-Schweitzer-Campus 1/Building A11, Münster 48149, Germany; Institute of Medical Data Science, Otto-von-Guericke University Magdeburg, Leipziger Str. 44/Building 2, Magdeburg 39120, Germany; Clinic for Cardiology II: Electrophysiology, University Hospital Münster, Albert-Schweitzer-Campus 1/Building A1, Münster 48149, Germany; Institute of Medical Informatics, University of Münster, Albert-Schweitzer-Campus 1/Building A11, Münster 48149, Germany; Clinic for Cardiology II: Electrophysiology, University Hospital Münster, Albert-Schweitzer-Campus 1/Building A1, Münster 48149, Germany

We thank the author for his constructive commentary on our work describing QTc estimation with QTcNet.^1^ We appreciate the recognition of both the external validation strategy and the open-source framework, and we agree that the following points highlight important methodological aspects for translating machine learning-based ECG analysis into clinical practice.

First, regarding the reported 15-ms correction observed in EDMS data, we concur that institution- and algorithm-specific differences cannot be generalized across settings. QTcNet was intentionally trained on heterogeneous datasets and evaluated externally to promote robustness across varying domains. Nevertheless, site-specific calibration is advisable prior to local clinical use. Additional analyses revealed a comparable systematic bias in the independent dataset EchoNext,^2,3^ in which ECG signals and QTc values were derived from the same GE Muse ECG management system. Future work will have to focus on recalibration protocols enabling standardized adaptation to new acquisition systems and institutional data characteristics.

Second, the reduced performance observed in atrial fibrillation and atrial flutter represents an important clinical limitation. Irregular RR intervals fundamentally complicate QT correction for both automated systems and expert readers. Thus, this subgroup should be explicitly considered during model training. Notably, prior work has shown that deep learning models trained specifically on atrial fibrillation cohorts can achieve reliable QT correction.^4^ Future work may explore an ensemble-of-experts framework that integrates specialized models for these subgroups. We overall recommend explicitly validating this subgroup in future models.

Third, the observation of regression-to-the-mean tendencies and the handling of outliers are also relevant. As discussed in our manuscript, this behaviour reflects both a strength and a limitation of the model.^1^ We support the suggestion that larger discrepancies between QTcNet predictions and conventional algorithmic estimates should be explicitly flagged. Such discrepancy-based safeguards should be reviewed carefully and considered specifically regarding risk-adapted human-in-the-loop framework.

Finally, we share the concern that performance gains from local fine-tuning may come at the cost of reduced external validity. In this context, distinguishing between global model development and local clinical deployment is crucial.^5^ As discussed above in relation to acquisition system-specific recalibration, fine-tuning can be considered as an analogous strategy when applied to adapt a model to local annotation conventions or system-specific characteristics. From this perspective, the reduced external performance following targeted fine-tuning reflects more of an expected trade-off rather than a fundamental limitation of the modelling approach. Consequently, the training strategy prioritized broad generalization, which explains why expert-driven fine-tuning on PTB data was not integrated into the global model. Nevertheless, we fully agree that domain adaptation techniques are promising for improving global model robustness while preserving cross-institutional performance. Future work should integrate larger amounts of expert-annotated data with carefully designed weighting schemes, alongside explicit domain adaptation strategies.

We are grateful for the thoughtful contribution to the discussion and for emphasizing clinically meaningful evaluation principles. Such dialogue is essential for ensuring that methodological advances translate into effective real-world applications.

## Data Availability

No new data were generated or analysed in support of this Letter.
